# Variable-angle total internal reflection fluorescence microscopy of intact cells of *Arabidopsis thaliana*

**DOI:** 10.1186/1746-4811-7-27

**Published:** 2011-09-24

**Authors:** Yinglang Wan, William M Ash, Lusheng Fan, Huaiqin Hao, Myung K Kim, Jinxing Lin

**Affiliations:** 1 Key Laboratory of Plant Molecular Physiology, Institute of Botany, Chinese Academy of Sciences, Beijing 100093, China; 2 Digital Holography and Microscopy Laboratory, Department of Physics, University of South Florida, Tampa, Florida 33620, USA; 3 Gradual School of Chinese Academy of Sciences, Beijing, 100049, China

**Keywords:** Quantitative, VA-TIRFM, optical analysis, intact cell, cell wall

## Abstract

**Background:**

Total internal reflection fluorescence microscopy (TIRFM) is a powerful tool for observing fluorescently labeled molecules on the plasma membrane surface of animal cells. However, the utility of TIRFM in plant cell studies has been limited by the fact that plants have cell walls, thick peripheral layers surrounding the plasma membrane. Recently, a new technique known as variable-angle epifluorescence microscopy (VAEM) was developed to circumvent this problem. However, the lack of a detailed analysis of the optical principles underlying VAEM has limited its applications in plant-cell biology.

**Results:**

Here, we present theoretical and experimental evidence supporting the use of variable-angle TIRFM in observations of intact plant cells. We show that when total internal reflection occurs at the cell wall/cytosol interface with an appropriate angle of incidence, an evanescent wave field of constant depth is produced inside the cytosol. Results of experimental TIRFM observations of the dynamic behaviors of phototropin 1 (a membrane receptor protein) and clathrin light chain (a vesicle coat protein) support our theoretical analysis.

**Conclusions:**

These findings demonstrate that variable-angle TIRFM is appropriate for quantitative live imaging of cells in intact tissues of *Arabidopsis thaliana*.

## Background

Total internal reflection fluorescence microscopy (TIRFM), also known as evanescent wave microscopy (EWM), is a powerful tool for observing the distribution and movement of fluorescently labeled molecules in an aqueous environment. This technique can be used when the molecules of interest are very close to the boundary between the aqueous environment and another medium with a higher refractive index (*n*). It is based on the physical phenomenon of total internal reflection (TIR), which occurs when a ray of light strikes a boundary between two materials with different *n *values and the incident angle (*θ_i_*) is greater than the critical angle of incidence (*θ_c_*) [1]. Under these conditions, all of the light is reflected back into the medium with the higher *n *value (**Formula 1**) [1]. When TIR occurs, a nearfield light wave forms at the boundary; this "evanescent wave" (EW) can penetrate the surface of the medium to a depth (*d*) approximately equal to 1/3 of the wavelength of the incident light (**Formula 2**) [1].

Formula for calculating critical angle of incidence:

(1) $$\theta c=\sin^{-1}\left(n_{1}∕n_{2}\right)$$

Formula for calculating the depth of an EW field:

(2) $$d=\lambda_{0}∕4\pi {\left(n_{2^{2}}\sin^{2}\theta -n_{1^{2}}\right)}^{-1∕2}$$

The TIRFM can provide a few hundred nanometers of excitation volume near the plasma membrane; this feature is particularly useful because the excitation volume is the site of many important events associated with the plasma membrane. TIRFM provides a higher spatial resolution and a higher signal-to-noise ratio than classic fluorescence microscopy or laser scanning confocal microscopy because the cytosol outside of the EW field remains unilluminated. When equipped with an electron-multiplier charge-coupled device (EM-CCD), the instrument can provide reliable imaging even with weak signals and a short exposure time. Therefore, the EM-CCD/TIRFM combination is a viable solution for laboratories; it provides high spatial and temporal resolution for studying events occurring near the plasma membrane [2-4].

Because the TIRFM technique is unrivaled in its imaging capability in the near-boundary field, it has been successfully applied to *in vivo *studies of animal cells for the past decade [3,4]. However, the thick cell wall that surrounds the cytoplasm of plant cells has cast doubt on the possible utility of TIRFM in plant cell studies [5,6]. Recently, a new technique known as variable-angle epifluorescence microscopy (VAEM) was developed to circumvent this problem. The technique allows laser light to penetrate the cell wall using a sub-critical angle in which *θ_i_*is slightly smaller than *θ_c_*[7], and it has been used successfully in studies of the membrane physiology of plant cells [8,9]. However, because VAEM provides a relatively deep illumination field of variable depth, it is sometimes referred to as pseudo-TIRFM [5].

In the present study, we coupled our TIRFM equipment to a device that was used to vary *θ_i_*. Using this technique of variable-angle TIRFM (VA-TIRFM), we were able to observe the dynamic and endocytotic behaviors of green fluorescent protein (GFP)-tagged plant membrane proteins in living *Arabidopsis thaliana *cells. The specific membrane proteins examined were phototropin 1 (PHOT1; a photoreceptor protein) and clathrin light chain (CLC; an endosomal vesicle coat protein). During our experiments, we observed that PHOT1-GFP and CLC-GFP both exhibited two different modes of dynamic behavior, a *lateral-movement mode *and a *blinking mode *respectively. Observation of the blinking mode occurred only with true TIRFM, which provides an EW field with a constant depth of illumination. Therefore, we carried out an optical analysis to explain this experimental phenomenon and investigated whether VA-TIRFM will prove useful in the quantitative analysis of membrane-associated proteins in intact plant cells.

## Results and discussion

### Optical analysis of plant peripheral layers and limitations of VAEM in plant-cell studies

**Figure 1:**
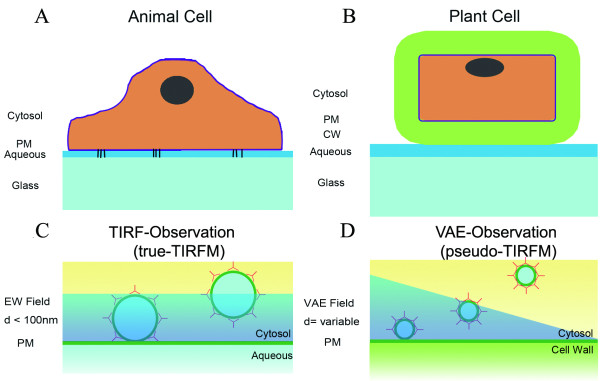
**Peripheral boundaries around animal and plant cells**. (A) Schematic model of peripheral boundaries around an animal cell migrating on a glass surface. (B) Schematic model of peripheral boundaries around an intact plant cell. (C) In cells without cell walls (animal cells or protoplasts of plant cells), a typical EW field with a constant depth of illumination forms in the cytosol. (D) A VAEM device can produce a variable depth of illumination in an intact plant cell. PM: plasma membrane; CW: cell wall; EW: evanescent wave; TIRFM: total internal reflection fluorescence microscopy; VAE: variable-angle epifluorescence.

**Figure 2:**
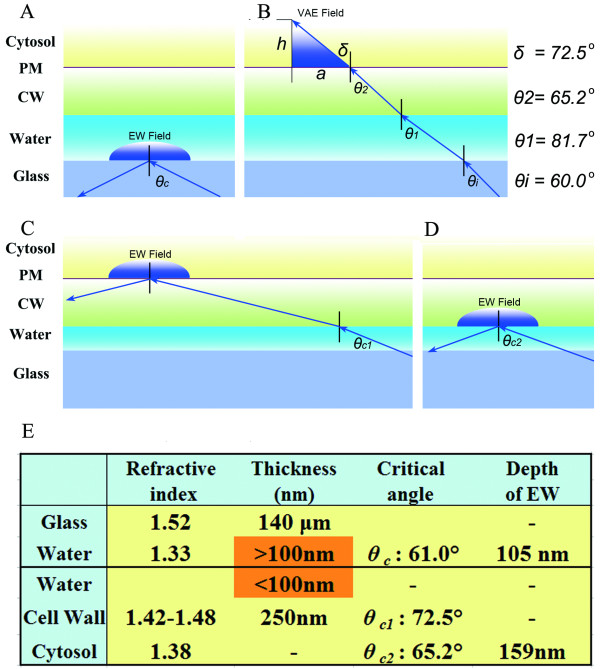
**Optical analysis of light paths in VAEM and TIRFM observations in plant cells**. (A) When the aqueous film between the cover glass and the cell wall is thick (> 100 nm) and *θ_i_*is greater than 61.0° (*θ_c_*), all light energy is reflected at the g/am interface. The EW field forms in the aqueous medium but cannot penetrate into the cytosol. (B) When *θ_i_*is less than 61.0° (*θ_c_*), a VAE field forms, illuminating the cytosol with variable depth. Angles of refraction for all optical boundaries with *θ_i_*= 60.0° are shown. (C) When the aqueous film is sufficiently thin (< 100 nm), and *θ_i_*is smaller than *θ_c_*for g/cw (*θ_c2_*) but greater than *θ_c_*for g/c (*θ_c1_*) [(*i.e*., *θ_c1_*<*θ_i _< θ_c2_*)], an EW field of constant depth is created inside the cytosol at the cw/c interface. (D) When *θ_i _> θ_c2_*, the light is completely reflected at the cell wall interface, and the EW field is not deep enough to penetrate into the cytosol. (E) Optical parameters of plant-cell peripheral boundaries. In the situation shown in (A), the EW field is 105 nm deep and cannot penetrate the cell wall. In the situation shown in (C) and with *θ_i _*= 67.0°, the EW field penetrates 159 nm into the cytosol. CW: cell wall; EW: evanescent wave; PM: plasma membrane; VAE: variable-angle epifluorescence.

To analyze the optical principles underlying our fluorescence observations of plant cells, we first examined the peripheral layers around the plant cytosol. Unlike microscopic observations of animal cells (Figure 1A), microscopic observations of plant cells (Figure 1B) must accommodate five different peripheral layers between the incident light and the cytosol (*n *= 1.38); these layers are the immersion oil (*n *= 1.52), glass cover slip (*n *= 1.52), aqueous medium (*n *= 1.33), cell wall (*n *= 1.42 to 1.48), and plasma membrane (*d *= 7 nm) [1,10]. This complex optical layering causes two major difficulties in the application of TIRFM technology to plant cell studies. First, because the aqueous layer between the cell wall and the cover slip has the smallest refraction index of these peripheral layers, the interface between the glass and the aqueous medium (g/am interface) has the smallest *θ_c_*of all the interfaces (Figure 2A). Second, because the cell wall is quite thick (> 200 nm) [11], the depth of the EW field (~100 nm) is insufficient to penetrate this thick boundary when TIR occurs at the g/am interface (Figure 2A). VAEM solves these problems by creating a sub-critical angle of incidence, resulting in a narrow illumination field inside cytosol Figure 1D and 2B) [7].

According to **Formula 1**, *θ_c_*at the g/am interface is 61.0°. The angle of refraction (*δ*) inside the cytosol for a sub-critical angle of incidence (*θ_i _< θ_c_*) can be calculated using Snell's law (**Formulas 3 **and **4**). With the *n *values given above for cytosol and glass and *θ_i_*set at 60.0° (slightly less than *θ_c_*), then *δ *is 72.5° inside the cytosol. The depth of the illumination field from the refractive laser light can then be calculated by a trigonometric formula (**Formula 5**).

***Snell's law ****describes the relationship between θ_i _and δ as:*

(3) $$\sin\theta_{i}∕\sin\delta =n_{2}∕n_{1}$$

When incident light penetrates × different optical layers, δ in the last medium (δ_x_) can be calculated as:

(4) $$\sin\theta_{i}∕\sin\delta_{x}=n_{x}∕n_{1}$$

Variable depth of illumination (d) within an observation field (width = a) can be calculated as:

(5) $$d=a∕\tan\delta_{x}$$

In our experimental set-up, the observation field of the EM-CCD camera is 512 × 512 pixels, equivalent to 81.9 × 81.9 μm (*a *= 81.9 μm); thus, according to **Formula 5**, *d *can vary from 0 to 25.9 μm in this field (Figure 2B and 2E). Given that the width of a single cell is 20 μm, *d *can vary from 0 to 6.3 μm. This theoretical result is consistent with the experimental results reported by Konopka *et al*., who used VAEM to observe GFP-tagged CLC and the GFP-tagged microtubule-binding domain in intact plant cells; they determined that the depth of the VAEM observation ranged from a few microns to approximately 40 microns [7]. Therefore, we have designated this illumination field as the variable-angle epifluorescence (VAE) field (Figure 1D), distinct from the EW field associated with TIRFM (Figure 1C).

The narrow and constant EW illumination field is the major advantage of the TIRFM technique over laser scanning or spinning-disc confocal microscopy [3]. On the other hand, VAEM does not exceed these techniques in spatial resolution and is markedly limited in its usefulness for observations of intact plant cells because the variable illumination depth prevents accurate quantitation. The observed molecules do not maintain a constant dynamic state between different observations or among the different regions in a single observation with a variable illumination depth. In contrast, true TIRFM can be used to quantitatively measure the protein docking time on the plasma membrane [12] and to analyze the oligomeric states of membrane proteins in animal cells [13]. Thus, VAEM is inferior to true TIRFM; the disadvantages outlined above limit its suitability for studies of protein dynamics and behaviors in plant cell membranes.

### "Frustrated" TIR and EW field in intact plant cells

Another major difference between plant and animal cells is that unlike migrating animal cells, plant cells do not adhere to glass surfaces. Thus, an aqueous film of varying thickness is present between the glass surface and the cell wall. When laser light strikes the g/am interface with *θ_i _*>*θ_c_*, and the aqueous film is sufficiently thin (*h *< 1/5 λ; approximately 100 nm), the tail of the evanescent wave transmits most of the light into the next medium so that only a small part of the light energy is reflected back; this phenomenon has been dubbed "frustrated" TIR (fTIR) [14]. After calculating the altered *θ_c_*and the loss of energy in the fTIR situation, Ash *et al*. used TIR holographic microscopy to examine onion epidermal cells attached to a glass surface [15]. Their analysis suggested that laser light can penetrate the aqueous medium even when *θ_i _*>*θ_c_*. The critical factor for the fTIR phenomenon is a sufficiently thin aqueous film.

As shown in Figure 2C and 2D, a laser light beam can penetrate the aqueous medium under fTIR conditions. Thus, the first major obstacle to the application of TIRFM to plant-cell studies is overcome. If we consider the *n *values of the different peripheral layers in plant cell microscopy, we see that TIR may occur at two different interfaces, the cell wall/cytoplasm (cw/c) interface and the glass/cell wall (g/cw) interface. Given that the average *n *of cell walls is 1.45 [10] and that cell walls of outer epidermal cells are smooth and of constant thickness (roughly 250 nm) [11], *θ_c_*at the cw/c interface (*θ*_*c(cw/c)*_) is 72.1°. According to Snell's law (**Formula 3**), *θ_i_*from the objective lens (*θ_c1_*in Figure 2C and 2E) should be greater than 65.2° to create TIR at the cw/c interface. When *θ_i_*is further increased by the variable angle device and reaches *θ_c_*at the g/cw interface (*θ*_*c(g/cw) *_= 72.5°), the light beam is completely reflected back (*θ_c2_*in Figure 2D). Again, the EW field cannot reach the cytosol in an intact cell. There are two critical requirements to obtain EW field illumination inside the cytosol in an intact plant cell: an incident angle between *θ_c1 _*and *θ_c2_*(65.2° *< θ_i_*< 72.5°) and a sufficiently thin aqueous film (*d *< 100 nm). When the EW field occurs inside the cytosol of an intact plant cell, the depth of illumination is constant for a single observation (Figure 2E).

Cell walls can be removed through enzymatic digestion to create protoplasts, "naked" plant cells that provide another approach for obtaining EW field illumination inside the plant cell cytosol. When protoplasts are tightly attached to a glass surface, the EW field can penetrate into the cytosol in a manner similar to that in TIRFM observation of animal cells (Figure 1C). However, the preparation of protoplasts seems likely to affect the organization of at least some membrane and cytoskeletal proteins [16,17], limiting the usefulness of protoplasts in physiological studies.

### EW and VAE field observations of PHOT1-GFP and CLC-GFP in intact plant cells

**Figure 3:**
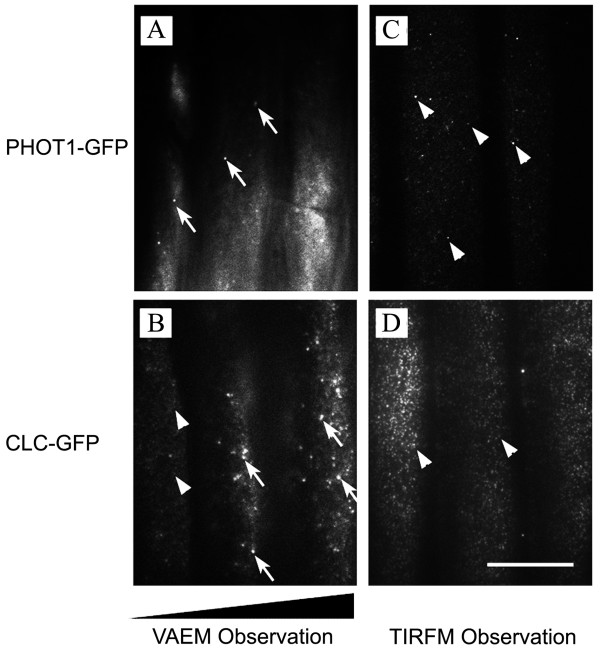
**PHOT1-GFP and CLC-GFP fluorescence observations by VAEM and TIRFM**. Still images taken from time-lapse sequences indicate localization of PHOT1-GFP (A and C) and CLC-GFP (B and D) in hypocotyl epidermal cells of 4-day-old *Arabidopsis *seedlings. Observations were made using VAEM (A, B) or TIRFM (C, D) observations. GFP-labeled pits in lateral-movement mode are indicated by arrows, and those in blinking mode are indicated by arrowheads. The shaded wedge in (B) indicates the trend of changing depth of VAE field illumination. TIRFM: total internal reflection fluorescence microscopy; VAEM: variable-angle epifluorescence microscopy. Bar = 20 μm.

To test our theoretical analysis of EW and VAE field observation, we performed TIRFM and VAEM experiments. The results were unambiguous and reproducible. For example, when we examined GFP-tagged PHOT1 or CLC in transgenic *Arabidopsis *cells using VA-TIRFM, we observed two different dynamic modes: a *lateral-movement mode *(Figure 3A and 3B), additional movie files show this mode in more detail (see Additional file 1** and **2** online**); and a *blinking mode *(Figure 3C and 3D), additional movie files show this mode in more detail (see Additional file 3 and 4** online**). Because a uniformly present blinking mode can only be observed in an EW field with a constant depth of illumination, our observation of a blinking mode was a true-TIRFM observation. (Actually, it was this original observation that prompted us to analyze the optical characteristics of the peripheral layers in plant-cell microscopy. In turn, these theoretical analyses of the fTIR phenomenon have supported our TIRFM observations in intact plant tissues).

PHOT1 is a membrane-associated receptor of blue light that localizes on the peripheral membrane of hypocotyl cortical and epidermal cells [18]. In 4-day-old dark-grown seedlings, PHOT1-GFP is found on the plasma membrane, but exposure to blue light initiates its internalization by activating its functional domains [18,19]. In our experiments, we found that the lateral-movement mode of PHOT1-GFP was observable only after a 3-5-min period of blue laser illumination (Figure 3A and Additional file 1 online), suggesting that the PHOT1-GFP spots moving laterally were blue-light-induced PHOT1-GFP endosomes moving inside the cell near the plasma membrane (*i.e*., in the VAE field).

When we changed the *θ_i_*so that *θ_c1 _< θ_i_*<*θ_c2_*(Figure 2C) and gently pressed the seedling against the surface of the glass slide, we observed a typical TIRFM image showing blinking behavior of the labeled protein (Figure 3C and Additional file 3 online). This finding confirms the theoretical analysis above, showing that with a sufficiently thin water film and a carefully adjusted *θ_i_*, membrane receptor proteins can also be observed in the EW field (Figure 2C). In comparing the dynamic modes of PHOT1-GFP molecules, we found that the PHOT1-GFP spots exhibiting lateral movement were both more numerous and more intense than those exhibiting blinking behavior. This observation suggested that some of the PHOT1-GFP was assembled in early endosomes or the *trans*-Golgi network, whereas the PHOT1-GFP in the plasma membrane was distributed as monomers or dimers (Kaiserli *et al*. 2009). Together, these observations of PHOT1-GFP provide solid evidence supporting our current functional model of VA-TIRFM observation in intact plant cells (Figure 2C).

This functional model was also confirmed by our observations of the dynamic modes of CLC-GFP in an intact *Arabidopsis *seedling. CLC molecules normally assemble together with clathrin heavy chains to form a "cage" that coats endosomal vesicles [20]. The docking time of CLC-GFP signals at the plasma membrane may reflect the maturation phase of clathrin-coated pits [12,21]. Because many CLC-GFP molecules interact during cage assembly on the plasma membrane, whereas PHOT1 exists only as monomers and dimers on the plasma membrane [19,20], the CLC-GFP and PHOT1-GFP spots were expected to be of different sizes and to reflect different dynamic behaviors (Figure 3). Nonetheless, as we observed for PHOT1-GFP, CLC-GFP also exhibited both blinking and lateral-movement modes. Interestingly, the blinking mode (arrowheads in Figure 3B) and the lateral-movement mode (arrows in Figure 3B) were both observed in the same visual field (Additional file 2** online**), indicating a highly variable depth for the VAE field. In the regions with more shallow illumination, most of the CLC-GFP spots were blinking, with only a few laterally moving spots present in the same cell (Figure 3B, *left*). In the regions with deep illumination fields, more laterally moving CLC-GFP spots were found (Figure 3B, *right*). In contrast, when cells were observed in a constant EW field, no laterally moving spots were detected in the entire visual field (Figure 3D and Additional file 4** online**). Taken together, these experimental results constitute strong evidence that true-TIRFM observations can be made in intact plant cells. Therefore, these observations are a valid basis for quantitative analyses.

## Conclusions

Our theoretical analyses and experimental results demonstrate that the *θ_i_*selected on VA-TIRFM equipment and the firmness of attachment of intact seedlings to the glass cover slip affect the type of observation of GFP-tagged membrane proteins that is made. Observation of lateral movement indicates VAEM, whereas observation of blinking behavior indicates a true TIRFM (EWM). These two clearly distinguishable results support the validity of quantitative analysis of VA-TIRFM results. Trajectory-tracking methods [22-24] can be used with VA-TIRFM to record the parameters of molecule movement, such as velocity and mean square displacement, allowing their roles in cellular trafficking and signaling processes to be analyzed. Furthermore, fluctuation in the observed fluorescence intensity might reflect movement through early endosomes or the *trans*-Golgi network from the plasma membrane [25]. Analyses of attachment time and photobleaching of blinking spots can be used to quantitatively determine the attachment mode, endocytosis rate, and oligomeric states of membrane-attached proteins [13,25,26].

## Methods

### Plant material

The Arabidopsis seedlings expressing *PRE_PHOT1_::PHOT1-GFP *in the phot1-5 background [27] and *PRE_CLC_::CLC-GFP *in WT (Col-0) background [8] were observed in this study. The seeds were sterilized and planted on the surface of 0.4% phytagel^® ^(**Sigma-Aldrich**, St. Louis, MO. USA) medium with 1/2 MS basal salts (**Sigma-Aldrich**) and 1% sucrose (**Sigma-Aldrich**). The seedlings were put in the chamber with illumination from white fluorescence lamps with 7000-10000 lux of illuminance for 16 hours per day at a temperature of 22°C.

### VA-TIRFM setup and observation

An objective-type TIR fluorescence microscope was built using an inverted microscope (IX-71, Olympus) equipped with a 100× oil-immersion objective lens (numerical aperture = 1.45) and a diode laser (Changchun New Industries Optoelectronics Tech. Co., Ltd., Changchun, China) for illumination of objects through an adjustable-angle internal reflective fluorescence illuminator. GFP-tagged molecules were excited by a 471 nm laser, respectively, and fluorescence signals were collected by the objective. Signals were filtered through either an FF01-525/45-25 band-pass filter (Semrock, Lake Forest, Illinois, USA) and then directed using a back-illuminated EM-CCD camera (ANDOR iXon DV8897D-CS0-VP, Andor Technology plc., Belfast, Northern Ireland). The Micro-Manager program (version 1.3; open source software developed by the Vale Lab at the University of California, San Francisco, CA, USA) was used to operate the equipment. For sample preparation, *Arabidopsis *seedlings were immersed in half-strength Murashige and Skoog liquid medium on a cover glass (BRAND Gmbh, Wertheim, Germany; *n*, 1.52 ± 0.01; thickness, 0.13-0.17 mm). Another cover glass was placed on top of the sample, and this sandwich was pressed gently to tightly attach the seedling to the glass surface.

## List of abbreviations

**CLC: **clathrin light chain; **CW: **cell wall; **cw/c: **cell wall/cytoplasm interface; **EM-CCD: **electron-multiplier charge-coupled device; **EW: **evanescent wave; **EWM: **evanescent wave microscopy; **fTIR: **"frustrated" TIR; **g/am: **glass/aqueous medium interface; **g/cw: **glass/cell wall interface; **GFP: **green fluorescent protein; **PHOT: **phototropin; **PM: **plasma membrane; **TIR: **total internal reflection; **TIRFM: **total internal reflection fluorescence microscopy; **VAE: **variable-angle epifluorescence; **VAEM: **variable-angle epifluorescence microscopy; **VA-TIRFM: **variable-angle total internal reflection fluorescence microscopy.

## Competing interests

The authors declare that they have no competing interests.

## Authors' contributions

YW performed the optical analysis, wrote the manuscript. LF and HH provided the experimental results. WMAIII and MKK proved the optical principles and provided important and essential suggestions on writing of this manuscript. JL supervised this project. All authors read and approved the final manuscript.

## Supplementary Material

Additional file 1 **All these additional video files are reconstructed from time lapse sequences with 300 images in a 60 fps frame rate**. The exposure time for each image is 100 ms, interval time between images is 50 ms. 1 pixel = 0.16 μm. VAEM observation of PHOT1-GFP (Pseudo-TIRFM). Click here for file

Additional file 2 **All these additional video files are reconstructed from time lapse sequences with 300 images in a 60 fps frame rate**. The exposure time for each image is 100 ms, interval time between images is 50 ms. 1 pixel = 0.16 μm. VAEM observation of CLC-GFP (Pseudo-TIRFM). Click here for file

Additional file 3 **All these additional video files are reconstructed from time lapse sequences with 300 images in a 60 fps frame rate**. The exposure time for each image is 100 ms, interval time between images is 50 ms. 1 pixel = 0.16 μm. TIRFM observation of PHOT1-GFP (True-TIRFM). Click here for file

Additional file 4 **All these additional video files are reconstructed from time lapse sequences with 300 images in a 60 fps frame rate**. The exposure time for each image is 100 ms, interval time between images is 50 ms. 1 pixel = 0.16 μm. TIRFM field observation of CLC-GFP (True-TIRFM). Click here for file
